# Pneumococal Surface Protein A (PspA) Regulates Programmed Death Ligand 1 Expression on Dendritic Cells in a Toll-Like Receptor 2 and Calcium Dependent Manner

**DOI:** 10.1371/journal.pone.0133601

**Published:** 2015-07-27

**Authors:** Mohit Vashishta, Naeem Khan, Subhash Mehto, Devinder Sehgal, Krishnamurthy Natarajan

**Affiliations:** 1 Infectious Disease Immunology Laboratory, Dr. B. R. Ambedkar Centre for Biomedical Research, University of Delhi, Delhi, India; 2 Molecular Immunology Laboratory, National Institute of Immunology, New Delhi, India; Instituto Butantan, BRAZIL

## Abstract

Pneumonia leads to high mortality in children under the age of five years worldwide, resulting in close to 20 percent of all deaths in this age group. Therefore, investigations into host-pathogen interactions during *Streptococcus pneumoniae* infection are key in devising strategies towards the development of better vaccines and drugs. To that end, in this study we investigated the role of *S*. *pneumoniae* and its surface antigen Pneumococcal surface protein A (PspA) in modulating the expression of co-stimulatory molecule Programmed Death Ligand 1 (PD-L1) expression on dendritic cells (DCs) and the subsequent effects of increased PD-L1 on key defence responses. Our data indicate that stimulation of DCs with PspA increases the surface expression of PD-L1 in a time and dose dependent manner. Characterization of mechanisms involved in PspA induced expression of PD-L1 indicate the involvement of Toll-Like Receptor 2 (TLR2) and calcium homeostasis. While calcium release from intracellular stores positively regulated PD-L1 expression, calcium influx from external milieu negatively regulated PD-L1 expression. Increase in PD-L1 expression, when costimulated with PspA and through TLR2 was higher than when stimulated with PspA or through TLR2. Further, knockdown of TLR2 and the intermediates in the TLR signaling machinery pointed towards the involvement of a MyD88 dependent pathway in PspA induced PD-L1 expression. Incubation of DCs with *S*. *pneumoniae* resulted in the up-regulation of PD-L1 expression, while infection with a strain lacking surface PspA failed to do so. Our data also suggests the role of PspA in ROS generation. These results suggest a novel and specific role for PspA in modulating immune responses against *S*. *pneumoniae* by regulating PD-L1 expression.

## Introduction

Pneumonia and pneumonia related illnesses are a major cause of mortality in children under the age of five years worldwide, with close to 20 percent of all deaths in this age group [1]. *S*. *pneumoniae* is the number one cause of bacterial pneumonia and can also cause meningitis, sepsis and otitis media. In some developing countries *Streptococcus pneumoniae* can account for over 50% of all pneumonia deaths [2, 3]. There are over 90 different capsular serotypes of *S*. *pneumoniae* based on the structure of the polysaccharide capsules [4]. A 7-valent pneumococcal conjugate vaccine (PCV7) is in use since 2000 and more recently a 13-valent pneumococcal conjugate vaccine (PCV13) has been licensed [5]. Pneumococci possess a number of virulence factors, including its polysaccharide capsule [6]. The capsule enables the pneumococci to evade entrapment by the mucus secretions that line the nasal cavity which the bacterium encounters initially. The capsule also helps protect pneumococci against opsonisation and killing by phagocytes [7]. In addition, *S*. *pneumoniae* also possess various surface associated proteins that contribute to its virulence; many of which are able to elicit measurable protection in mice e. g. Pneumococcal surface protein A (PspA) [6, 8, 9].

PspA, a serologically variable, cross-reactive, cross-protective protein is present on almost all strains of pneumococci and has been shown to be a promising candidate antigen for a protein-based vaccine [10–12]. Antibodies to PspA have been shown to protect mice from challenge when given passively [12–14]. PspA interferes with fixation of complement C3 on the pneumococcal surface [15], and its lactoferrin-binding activity is believed to protect pneumococci from bactericidal activity of apolactoferrin [16]. Further, the basic molecular structure of PspA is conserved in most pneumococcal strains [17]. Based on its sequence PspA has been classified in 3 families and 6 clades [18, 19]. Recently, it has been demonstrated that protection against vast majority of pneumococcal strains can be achieved by combining PspAs from different families or clades [20, 21]. Although PspA exhibits structural variability it posessess enough epitopes that are common to all pneumococci to confer protection against sepsis [22].

The costimulatory molecule Programmed Death Ligand-1 (PD-L1) is constitutively expressed and its surface expression is upregulated on murine hematopoietic cells (e. g., T cells, B cells, macrophages, dendritic cells and bone-marrow-derived mast cells) and non-hematopoietic cells (e. g., endothelial, epithelial and muscle cells) upon stimulation. It has been documented that PD-L1 interacts specifically with B7-1 to inhibit T cell proliferation [23]. The interactions of PD-1 with PD-L1 induce T cell inhibition and anergy, thereby terminating or preventing a productive T cell response and is generally considered as an immune inhibitory molecule. Consequently, many pathogens that cause both acute and chronic infections modulate the expression of these molecules on antigen presenting cells and T cells to their advantage. A number of infection studies with viral and fungal pathogens reported that PD-1:PD-L1 interactions inhibit T and B cell proliferation [24, 25] and blocking these interactions significantly rescues T cell functions and host resistance to infections [26]. Conversely, however, some studies also report that PD-1:PD-L2 (B7-DC) interactions can drive the proliferation of CD4^+^ and CD8^+^ T cells [27]. The PD-1: PD-L1 axis has been shown to be important for bacterial pathogens as well. PD-1:PD-L1 interaction is critical for the *Staphylococcus aureus* triggered conversion of neonatal CD4^+^ T cells into FOXP3^+^ CD25^+^ CD127^low^ T cells [28]. *S*. *aureus* modulates T cell responses via TLR2 and PD-L1 [29]. T cells from PD-L1^-/-^ mice respond inadequately to *Mycobacterium tuberculosis* infection [30]. PD-L1 expressed on human neutrophils infected with *Burkholderia pseudomallei* impairs T cell function [31].

DCs are crucial for innate and adaptive immune response against bacterial pathogens. In recent years, several groups have studied how pneumococcal virulence factors modulate DC function. The precise role of PD-1:PD-L1 pathway in dampening the T cell response is still unclear in pneumococcal infections. The role of PavA during pneumococcal infection has been investigated and it was demonstrated that it is essential for *S*. *pneumoniae* to escape phagocytic killing by DCs and for eliciting optimal proinflammatory cytokine production [32]. *S*. *pneumonniae* induces apoptosis in bone marrow derived DCs via two mechanistically different pathways. Pneumolysin dependent but caspase independent and delayed onset but caspase dependent mechanism accompanied with DC maturation [33]. Further, it has also been shown that alveolar DCs facilitate dissemination of *S*. *pneumoniae* during pneumococcal pneumonia [34]. Cao et al showed that Clp expression helps in the uptake of *S*. *pneumoniae* by DCs [35]. Clp could enhance programmed cell death in DCs that have phagocytosed pneumococci. Littmann et al reported that pneumolysin dampens the immune responses by specifically preventing human DC-mediated inflammatory responses [36]. However, the role of *S*. *pneumoniae* or its antigens in modulating PD-L1 expression on DC is not reported. In this study we investigated the role of *S*. *pneumoniae* and its antigen PspA in regulating the surface expression of PD-L1 on DCs. As DCs are known for their critical role in regulation and modulation of the host immune response against bacterial infections, in this study, we characterized the role of *S*. *pneumoniae* infection in regulating PD-L1 expression. Our data indicate that PspA induces the expression of PD-L1 on mouse bone marrow derived DCs. Results also point towards a role for the route of calcium entry and Toll-Like Receptor (TLR) 2 in mediating PD-L1 expression.

## Materials and Methods

### Ethics statement

Animal experiments were done with approval and in strict accordance with the guidelines of the National Institute of Immunology Institutional Animal Ethics Committee (IAEC#327/13). The procedures used in the study were carried out by well-experienced competent workers trained to perform them. All efforts were made to minimize suffering and pain.

### Bacterial strains and infection

Virulent *S*. *pneumoniae* strain D39 (capsular type 2), R6 (ATCC BAAA-255) and JY2008 (ATCC 55143) were used in the study [37]. JY2008 is a PspA mutant of Rx1 a non-capsulated descendent of D39. PspA is absent on the pneumoccocal surface as the 27 kDa truncated version of PspA (60% shorter than the full length PspA) expressed by JY2008 lacks the proline and repeat/anchor region that helps it in anchoring to the pneumococcal surface. As a result, the 27 kDa truncated PspA is released in small but detectable amounts in the culture supernatant. All strains were grown as described earlier [38] and maintained at the National Institute of Immunology. DCs were infected with live *S*. *pneumoniae* for 3h followed by addition of 40 μg/ml of gentamycin (to kill residual bacteria) and DCs were incubated at 37°C for an additional 24h.

### Materials

Phycoerythrin-tagged antibody against mouse PD-L1 were procured from BD Biosciences USA. Recombinant mouse Granulocyte macrophage colony stimulating factor (GM-CSF) was procured from R & D Systems (USA). Antibodies to pAkt, Bcl2, Bax and β-actin, specific and control siRNAs against mouse genes, and Luminol kit for chemiluminescence detection were purchased from Santa Cruz Biotechnology, USA. Chemicals U0126, 3, 4, 5-trimethoxybenzoic acid 8-(diethylamino)octyl ester (TMB-8), ethyleneglycol-bis(2-aminoethylether)-N, N, N', N'-tetraacetic acid (EGTA) and diphenyleneiodonium (DPI) were purchased from Sigma Chemical Co., USA.

### Dendritic cell enrichment and tranfection of siRNAs

Six to eight week old female BALB/c mice reared in a pathogen-free environment were used throughout the study. DCs were differentiated from mouse bone marrow as described previously [39]. Briefly, BALB/c mice were sacrificed by cervical dislocation, bone marrow from the tibias and femurs were flushed out, and lymphocytes and I-A^+^ cells were depleted following Magnetic Assisted Cell Sorting. Cells were cultured in RPMI 1640 medium containing 10% fetal bovine serum, 0.05 M 2-mercaptoethanol, 1 mM sodium pyruvate plus 15 ng/ml GM-CSF for 72h. Recombinant PspA was expressed and purified as described before [38]. The purity of PspA preparation found to be 96–98% as judged by Coomassie blue stained SDS-PAGE (S1 Fig). Unless mentioned otherwise DCs were stimulated with 15 μg/ml PspA for 24h. For some experiments, DCs were stimulated with various TLR ligands (TLR2 was stimulated with 1.0 μg/ml Pam_3_Csk_4_, TLR4 was stimulated with 0.1 μg/ml lipopolysaccharide (LPS) and TLR7 was stimulated with 1.0 μg/ml immiquimod) in the presence or absence of 15 μg/ml PspA for 24h. For some experiments, DCs were incubated with biopharmacological inhibitors to various molecules. For example, DCs were incubated with 100 μM TMB-8 for inhibiting calcium release from intracellular stores, 3 mM EGTA for inhibiting calcium influx from external medium, 10 μM U0126 for inhibiting MAPK-ERK and 10 μM diphenyleneiodonium (DPI) for inhibiting reactive oxygen species (ROS). For some experiments, DCs were transfected with siRNAs to various genes as described before prior to stimulation with 15 μg/ml PspA [40]. Briefly, bone marrow precursors (4 x 10^6^/ml) were transfected with 60 pmol of siRNA for 72h using the Hiperfect transfection reagent (Qiagen, Germany) in OPTIMEM medium (Invitrogen, USA). GM-CSF was added 5h following transfection, and incubation was continued for 72h for DC differentiation. Knockdown was verified by semi-quantitative reverse transcription-polymerase chain reaction. S2 Fig shows the knockdown efficiency of different genes relative to β-actin. At the end of the incubation period cells were processed either for flow cytometry or immunoblotting with for various molecules.

### Flow cytometry

At the end of the incubation period, DCs were first incubated with Fc-Block (BD Biosciences, USA) to mask CD16/CD32 Fc receptors and stained for the surface expression level of PD-L1 using phycoerythrin-tagged monoclonal antibody to PD-L1 and analyzed by flow cytometry (FACS Calibur, BD Biosciences) as described previously [39, 40]. The data were plotted and analyzed using CellQuest Pro software. No gates were applied.

### Confocal microscopy

DCs (2 x 10^6^/ml) were incubated with recombinant PspA^3–286^ from R36A [38]. Cells were fixed with 2% paraformaldehyde, permeabilized with 0.1% saponin, and incubated with antibodies against PD-L1 tagged with phycoerythrin. Cells were fixed with 4% paraformaldehyde. Confocal imaging was performed with Nikon C2 laser scan confocal microscope with 60x objective magnification, numerical aperture 1.4, refractive index 1.5, Plan Apo optics equipped with an argon laser, using excitation and emission wavelength of 405 and 488 nm, respectively. Data were analyzed using the NIS Elements AR software.

### Western blotting for signaling molecules

Immunoblotting was performed as described previously [40]. Briefly, at the end of incubation, cells were chilled on ice, washed once with ice-cold PBS and lysed in buffer containing 10 mM HEPES (pH 7.9), 10 mM KCl, 0.1 mM EDTA, 0.1 M EGTA, 0.5% Nonidet P-40, and 2.0 μg/ml of aprotinin, leupeptin and pepstatin each. The suspension was centrifuged at 18000 x g for 2 min at 4°C. The supernatant was designated as the cytoplasmic extract. The cytoplasmic extract (20 μg/ml) was resolved on 12% SDS-PAGE and transferred onto a nitrocellulose membrane (Hybond C pure, Amersham Biosciences, USA). The blots were probed with antibodies to various molecules, followed by appropriate horse radish peroxidase-labelled secondary antibodies. Furthermore, a parallel set of samples was run separately on SDS-PAGE and probed for β-actin as the loading control. The blots were developed by chemiluminescence using the luminol reagent.

### RNA isolation and reverse transcription-polymerase chain reaction

Total RNA was isolated from bone marrow derived DCs by TRIZOL method as described earlier [39, 40]. RNA was subjected to semi-quantitative RT-PCR to determine the transcript levels using the One-step RT-PCR kit (Qiagen) as per the manufacturer’s instructions. Semi-quantitative RT-PCR was carried out on a Bio-RAD MyCycler. The following primer pairs were used: for mouse PD-L1 forward 5′-CTAACAGGTGATCCGTTTCCTATG- 3′ and reverse 5′- GCCGTGATAGTAAACGCTGAA-3′ at 50°C for 30 min, 95°C for 15 min, 90°C 1 min, 1 min at 51°C, 72°C 1 min; for mouse β-actin forward 5′ TGTTACCAACTGGGACGACA- 3′ and reverse 5′-AAGGAAGGCTGGAAAAGAGC-3′ at 95°C 1 min, 60°C 1 min and 72°C 1 min. The resulting PCR products were separated and visualized on 1% agarose gel.

### Statistical analysis

A two tailed Student’s t-test was done to calculate *p* values. Values of *p* < 0.05 were considered as significant. Mean Fluorescence Intensities of each group were used for comparison and applied to Student’s t test *, *p≤ 0*.05; **, *p<0*.01 and ***, *p<0*.*001*.

## Results

### PspA induces PD-L1 expression on dendritic cells

**Fig 1:**
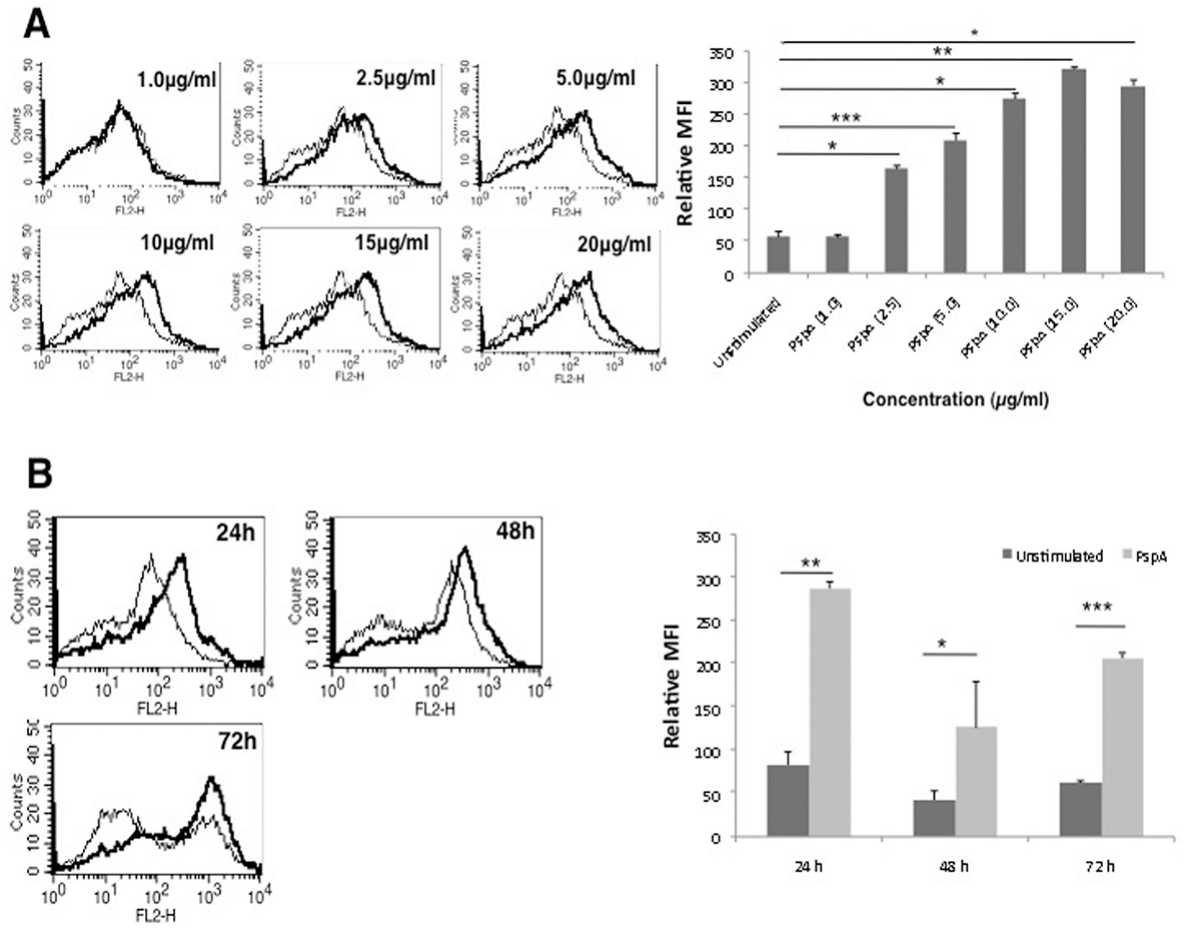
PspA upregulates expression of PD-L1 on DCs. A, mouse bone marrow derived DCs were stimulated with indicated concentrations of PspA for 24h. B, DCs were stimulated with 15μg/ml PspA for indicated times. PD-L1 expression was monitored by flow cytometry. Bold lines represent expression levels in the presence of PspA while thin lines represent unstimulated controls. Data from one of three independent experiments are shown. The MFI values from three independent experiment (expressed as mean ± SD) are summarized as a bar graph in the right panel.

We determined the surface expression of PD-L1 on DCs following stimulation with PspA using flow cytometry. As shown in Fig 1A and 1B, stimulation of DCs with PspA increased surface expression of PD-L1 in a dose and time dependent manner with maximum expression at 24h with 15 μg/ml. S3 Fig shows the specificity of staining with isotype control. Unless otherwise mentioned DCs were stimulated with 15 μg/ml for 24h for all subsequent experiments. The data indicated that PspA positively modulated the expression of PD-L1 on mouse bone marrow derived DCs. Bar charts next to each panel in Fig 1 represent the MFIs (mean ± SD) of three experiments.

### TLR2 synergizes with PspA to upregulate PD-L1

**Fig 2:**
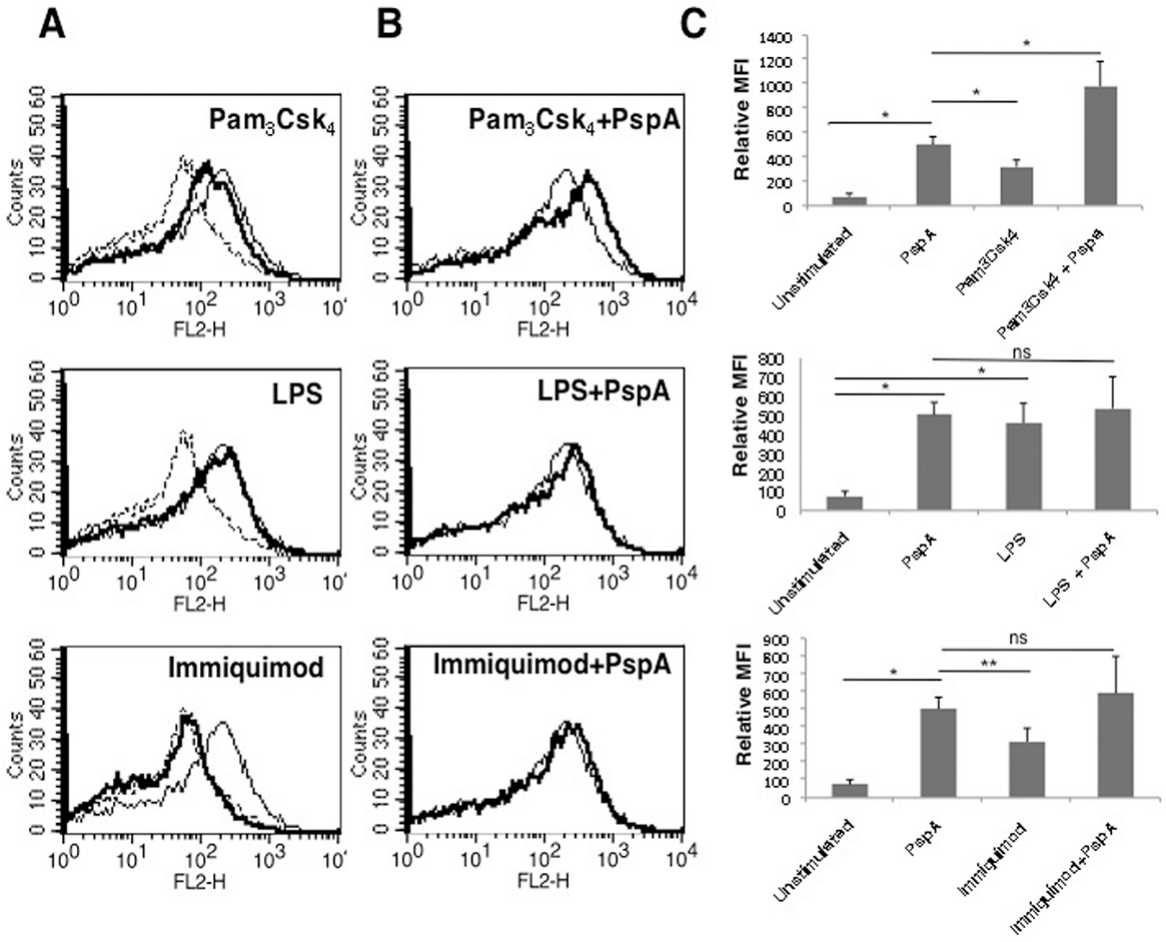
TLR2 and PspA synergistically stimulate PD-L1 up regulation. For Panel A, mouse bone marrow derived DCs were stimulated with either 15 μg/ml PspA, 1 μg/ml Pam_3_Csk_4_, 0.2 μg/ml LPS or 1 μg/ml Immiquimod for 24h and surface expression of PD-L1 was monitored by flow cytometry. Dotted lines represent unstimulated controls while thin lines represent stimulations with PspA. Bold lines represent stimulations with indicated agonists to different TLRs. Panel B, DCs were stimulated with 15 μg/ml PspA in the presence or absence of ligands to different TLRs for 24h and PD-L1 expression was monitored by flow cytometry. Thin lines represent stimulations with PspA alone. Bold lines represent costimulations with PspA and indicated agonists to different TLRs. Data from one of three independent experiments are shown. In Panel C, PD-L1 expression is represented as bars indicating fold change in Relative Mean Fluorescence Intensity (MFI) for various groups. Bars represent mean ± SD of three independent experiments. ‘ns’ represents non-significant differences between compared groups.

In order to characterize the molecular players involved in PspA mediated PD-L1 expression, we examined antigen receptors and key molecules involved in various signaling pathways that regulate PD-L1 expression. To investigate the role of pathogen associated receptors, we stimulated DCs with PspA in the context of costimulation with different TLR ligands or agonists. For example, we stimulated TLR2 with Pam_3_Csk_4_, TLR4 with LPS and TLR7 with immiquimod, along with PspA, and monitored the surface expression of PD-L1 on DCs by flow cytometry. As shown in Fig 2A, stimulation of DCs with TLR2 ligand Pam_3_Csk_4_ upregulated PD-L1 but to a lesser extent when compared with levels observed upon PspA stimulation, indicating that TLR2 stimulation also regulates PD-L1 expression on DCs. Interestingly, stimulation of TLR4 with LPS upregulated PD-L1 expression to the same levels as that observed with PspA. This indicated that lipopolysaccharides from Gram negative bacteria also upregulate PD-L1 on DCs. However, no upregulation of PD-L1 was observed upon stimulation of TLR7 with immiquimod indicating a minimal role for TLR7 in PD-L1 expression. Interestingly, costimulation of TLR2 with PspA increased PD-L1 expression over and above that obtained with PspA alone (Fig 2B and 2C, upper panel). However, no such synergy was observed when PspA was costimulated with either TLR4 or TLR7 (Fig 2B and 2C middle and lower panels). This indicated that PspA synergized specifically with TLR2 to upregulate PD-L1 expression. To further confirm the role of TLR2 in PspA induced PD-L1 expression in DCs, we knockdown TLR2 using specific siRNAs followed by stimulation of DCs with PspA. As shown in S4 Fig, knockdown of TLR2 significantly inhibited PspA induced PD-L1 upregulation clearly indicating a determinant role for TLR2 in PD-L1 expression.

### MyD88, IRAK1, TRAF6 and IRAKM regulate PD-L1 expression by TLR2 and PspA

**Fig 3:**
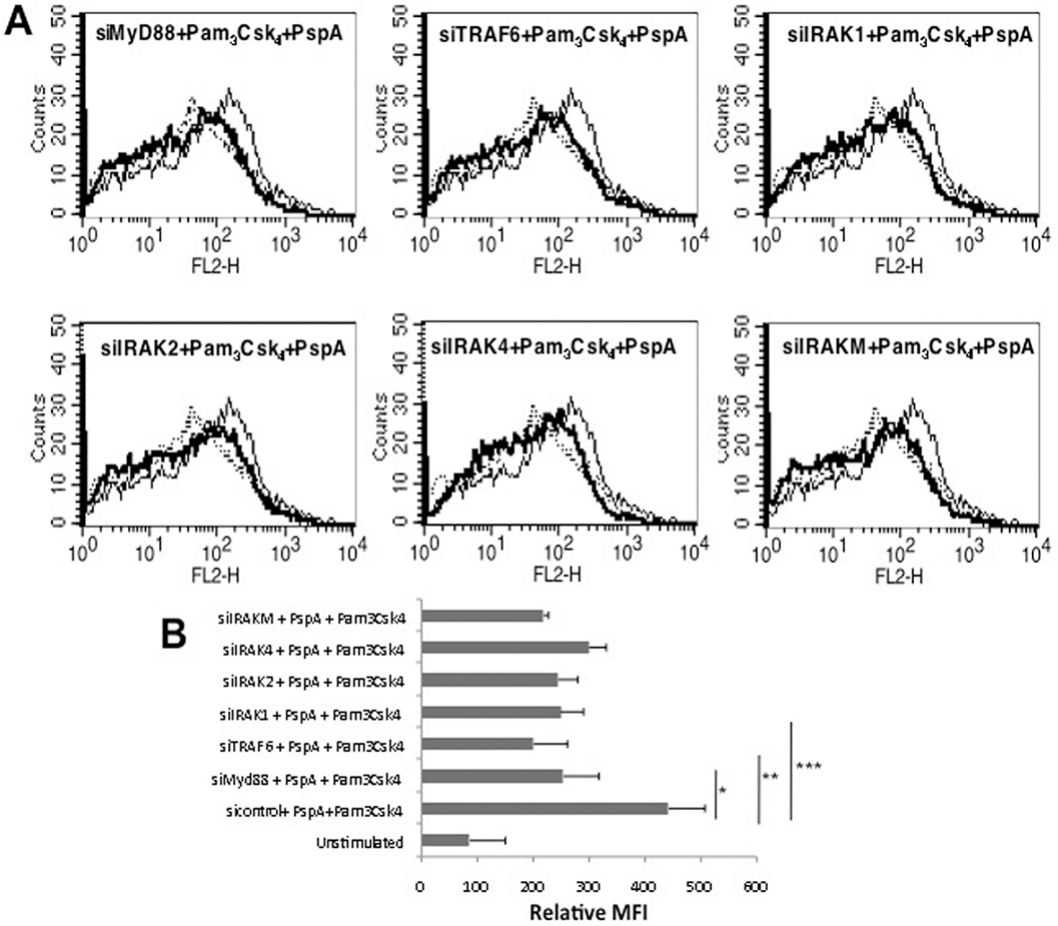
PspA induces PD-L1 expression in a MyD88 dependent pathway. Mouse bone marrow derived DCs were transfected with siRNAs against indicated molecules for 36h followed by stimulations with 1 μg/ml Pam_3_Csk_4_ and 15 μg/ml PspA for 24h. PD-L1 levels were monitored by flow cytometry. Dotted line represents unstimulated cells transfected with control siRNAs. Thin lines represent cells transfected with control siRNAs followed by stimulations with 1 μg/ml Pam_3_Csk_4_ and 15 μg/ml PspA. Bold lines represent cells transfected with specific siRNAs to indicated molecules followed by stimulations with 1 μg/ml Pam_3_Csk_4_ and 15 μg/ml PspA. Data from one of three independent experiments are shown. In Panel B, PD-L1 expression is represented as bars indicating fold increase in Relative Mean Fluorescence Intensity (MFI) for various groups. Bars represent mean ± S.D. of three independent experiments.

In order to identify the signaling components in the TLR pathway that regulate PspA induced PD-L1 expression, using specific siRNAs we knockdown major players in the pathway prior to PspA stimulation. As shown in Fig 3, knockdown of either MyD88, IRAK1, IRAK2, IRAK4, TRAF6 or IRAKM inhibited upregulation of PD-L1 expression following costimulation with Pam_3_Csk_4_ and PspA with maximum inhibition obtained upon knockdown of TRAF6. This indicated a role for MyD88 dependent pathway (summarized in Fig 3B) in PspA mediated PD-L1 expression and also confirmed the role of TLR2 in PspA induced PD-L1 expression.

### Route of calcium entry differentially regulates PspA mediated PD-L1 expression in DCs

**Fig 4:**
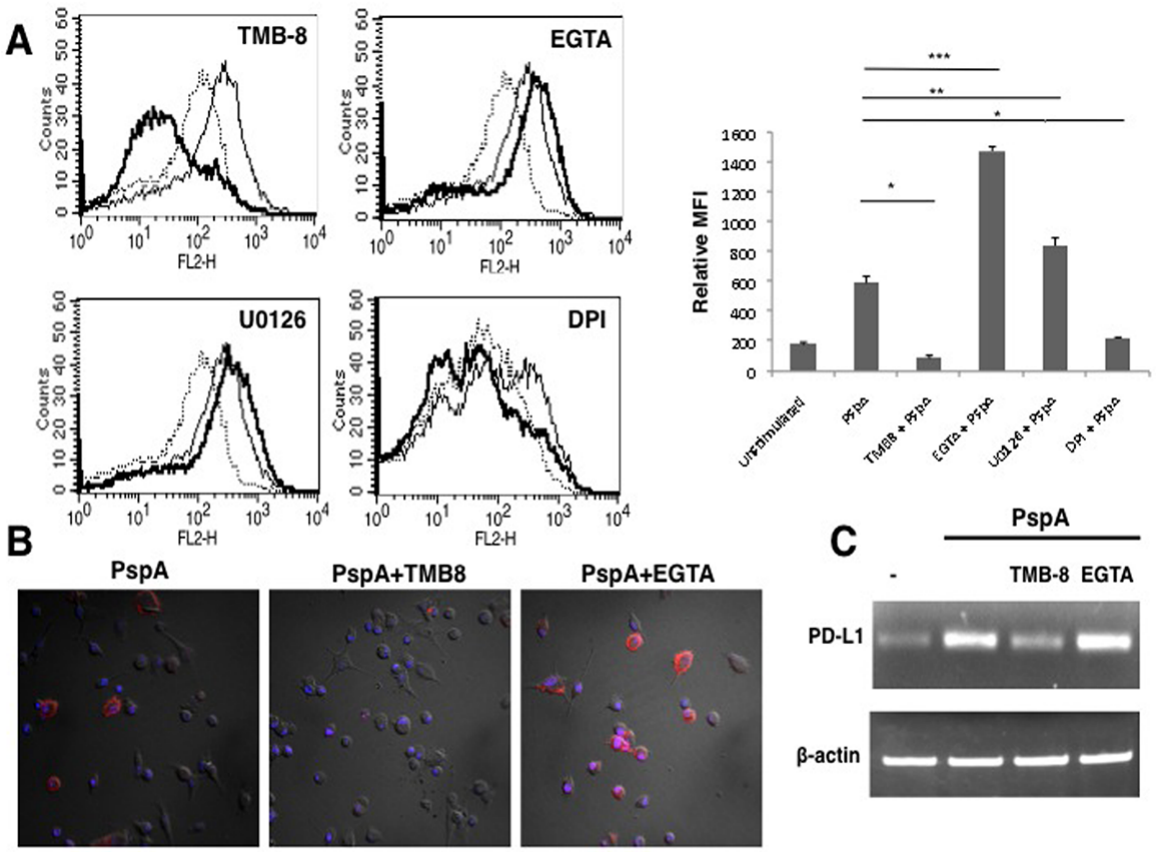
Route of calcium entry differentially regulates PspA induced PD-L1 expression. Mouse bone marrow derived DCs were incubated with bio-pharmacological inhibitors to indicated molecules for 1h followed by stimulation with 15 μg/ml PspA for 24h. PD-L1 levels were monitored by flow cytometry. Dotted lines represent unstimulated cells. Thin lines represent cells stimulated with PspA. Bold lines represent cells treated with inhibitors to indicated molecules followed by stimulation with PspA. One of three independent experiments is shown. PD-L1 expression is represented as bar graph indicating fold increase in Relative Mean Fluorescence Intensity (MFI) for various groups. Bars represent mean ± SD of three independent experiments. For Panel B, mouse bone marrow derived DCs were incubated in the presence or absence of TMB-8 or EGTA for 1h followed by stimulations with 15 μg/ml PspA for 24h. Cells were incubated with phycoerythrin conjugated anti-mouse PD-L1 antibody. Merged images with DAPI (blue) and PD-L1 (red) staining are depicted. For Panel C total RNA was isolated from cells stimulated as indicated and PD-L1 transcript levels were measured by semi-quantitative RT-PCR. One of three independent experiments is shown.

As signals from TLRs feed into multiple pathways, we next examined the possible role of key second messengers in these pathways to modulate PspA induced PD-L1 expression. To that end, using biopharmacological inhibitors, we investigated the role of calcium ion homeostasis, Protein Kinase C (PKC), Mitogen Activated Protein Kinase-Extracellular-signal Regulated Kinase (MAPK-ERK), PhosphatidylInositide-3 Kinase (PI-3K), Protein Kinase A (PKA), Calcium/calmodulin dependent protein Kinase II α (CAMKIIa) during PspA induced PD-L1 expression. To inhibit calcium homeostasis, we treated DCs with EGTA to chelate extracellular calcium and TMB-8 to inhibit intracellular calcium release from the endoplasmic reticulum. Interestingly, inhibiting intracellular calcium burst completely inhibited PspA induced PD-L1 expression, while inhibiting calcium influx from external milieu further increased PspA induced PD-L1 expression (Fig 4A). This indicated that the route of calcium entry in cells played a diverse and differential role in regulating PspA mediated PD-L1 expression. Co-inhibition of both internal calcium release and calcium influx from external stores inhibited PspA induced PD-L1 expression (data not shown). This indicated a dominant role for internal calcium release in regulating the expression of this co-stimulatory molecule.

Inhibiting ERK-MAPK with U0126 increased PspA induced PD-L1 expression suggesting the involvement of this pathway in PD-L1 expression (Fig 4A). On the other hand inhibiting either PKC or PI-3K or PKA did not significantly modulate PD-L1 expression indicating a minimal role for these molecules in PspA mediated PD-L1 expression (data not shown). We also investigated the role of Reactive Oxygen Species (ROS) in modulating PD-L1 expression, since our earlier work established a role for ROS in influencing immune responses as well as modulation of calcium dynamics in DCs during *M*. *tuberculosis* infection [40]. As shown in Fig 4A, inhibiting ROS with DPI completely inhibited PspA induced PD-L1 expression. This indicated that similar to calcium homeostasis from internal stores, ROS also played a positive role in regulating PspA induced PD-L1 expression. The overall results are summarized as a bar chart. We further investigated whether PspA modulated ROS levels in DCs. As shown in S5 Fig, stimulation of DCs with PspA significantly upregulated ROS levels. These data are in concurrence with the effect of PspA on PD-L1 expression and establish a direct relationship of ROS with PD-L1.

We further confirmed the above results on the requirement of calcium for PD-L1 expression by confocal microscopy. As shown in Fig 4, inhibiting IP_3_R with TMB-8 completely abrogated PspA induced PD-L1 expression, while inhibiting calcium influx from extracellular medium using EGTA increased PD-L1 expression (Fig 4B). These observations are in agreement with the data obtained in Fig 4A. Similar results were obtained for RNA levels of PD-L1, wherein stimulation with PspA increased PD-L1 transcript levels when compared to the control (Fig 4C). Further, calcium dynamics also similarly modulated PD-L1 transcript levels wherein treatment with TMB-8 decreased PspA induced PD-L1 mRNA levels while treatment with EGTA further increased PD-L1 transcript levels (Fig 4C).

### Molecular sensors of calcium homeostasis regulate PspA induced PD-L1 expression

**Fig 5:**
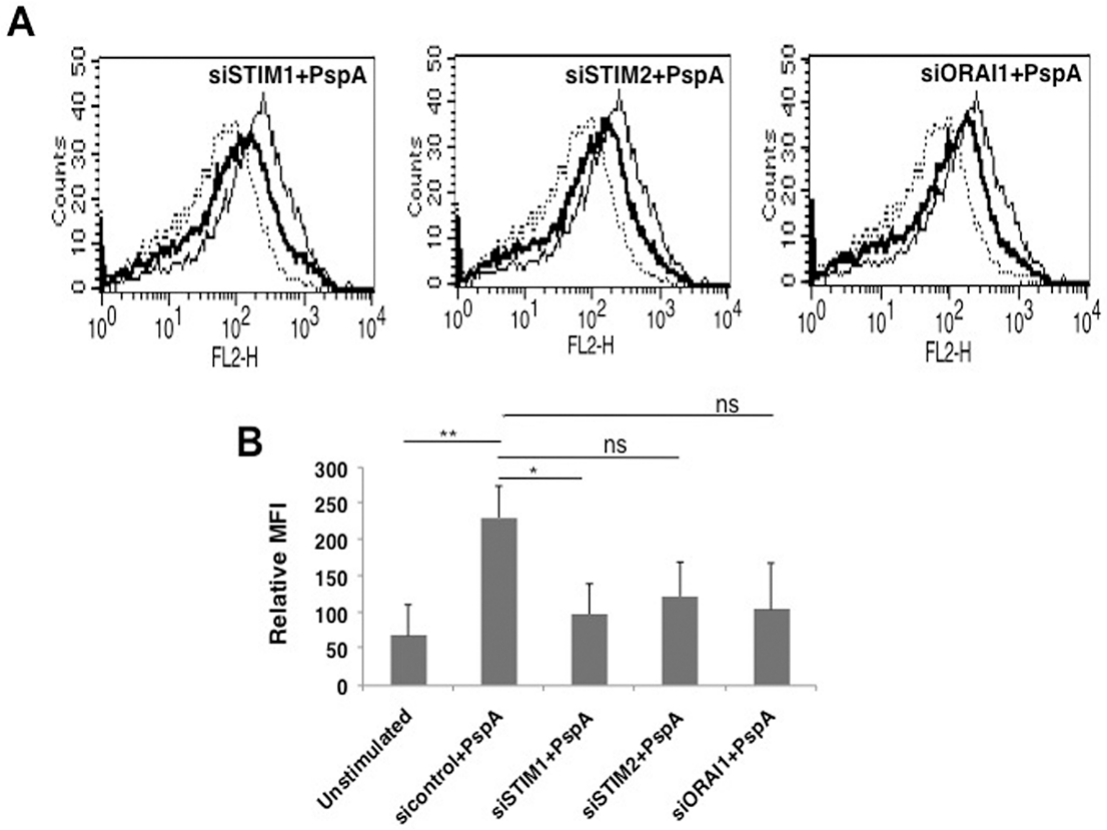
Molecular sensors of intracellular calcium regulate PspA induced PD-L1 expression. For Panel A, mouse bone marrow derived DCs were transfected with siRNAs against indicated molecules for 36h, followed by stimulation with 15 μg/ml PspA for 24h. PD-L1 levels were monitored by flow cytometry. Dotted lines represent unstimulated cells transfected with control siRNAs. Thin lines represent cells transfected with control siRNAs followed by stimulations with 15 μg/ml PspA. Bold lines represent cells transfected with siRNAs specific to the indicated molecules followed by stimulation with 15 μg/ml PspA. Data from one of three independent experiments is shown. In Panel B, PD-L1 expression is represented as bars indicating fold increase in Relative Mean Fluorescence Intensity (MFI) for various groups. In panel B, bars represent mean ± SD of three independent experiments. ‘ns’ represents non-significant differences between compared groups.

We further dissected the role of calcium homeostasis in regulating PspA mediated PD-L1 expression by investigating the role of molecular sensors of calcium ion concentration inside cells. To that end, we investigated the role of STromal Interaction Molecule (STIM) proteins STIM1 and STIM2, the molecular sensors that regulate Store Operated Calcium Entry (SOCE) via the Calcium Release Calcium Activated Channel (CRAC) ORAI1. As shown in the Fig 5, knockdown of STIM1 and to an extent (STIM 2 and ORAI1) significantly inhibited PspA mediated PD-L1 expression. This indicated that members of SOCE pathway regulate PD-L1 expression in DCs upon PspA stimulation. These results further substantiated the critical role for calcium dynamics in regulating PD-L1 expression during *S*. *pneumoniae* infection. To the best of our knowledge this is the first report of the involvement of SOCE channels in regulating PD-L1 expression during *S*. *pneumoniae* infection.

### *S*. *pneumoniae* induced upregulation of PD-L1 expression is calcium dependent

**Fig 6:**
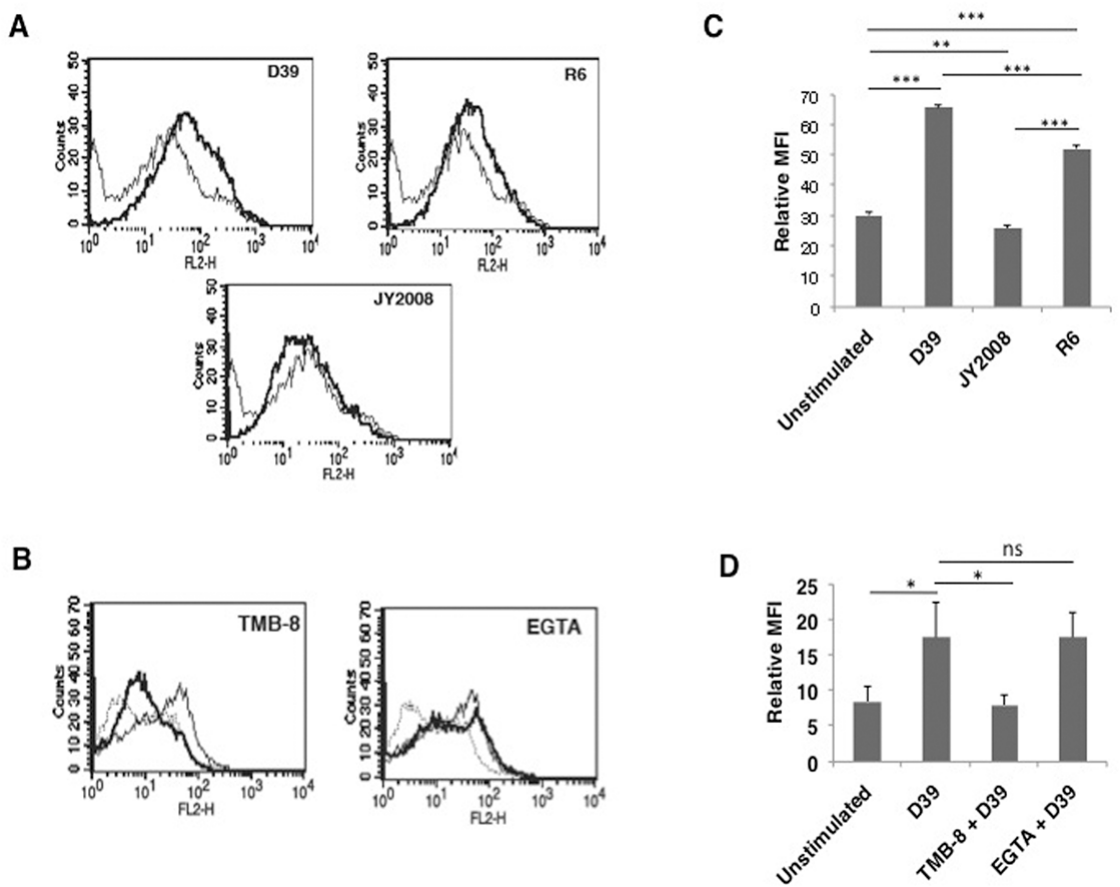
*S*. *pneumoniae* upregulates PD-L1 on DCs. For Panel A, mouse bone marrow derived DCs were infected with wild typ*e S*. *pneumoniae* strain D39, R6 or JY2008 (*see* Materials and Methods) and PD-L1 levels were monitored by flow cytometry. Thin lines represent uninfected cells while bold lines represent cells incubated with indicated strain of *S*. *pneumoniae*. For Panel B, DCs were incubated with either TMB-8 or EGTA for 1h followed by incubation with D39 and PD-L1 levels were monitored by flow cytometry. Dotted line represents uninfected cells. Thin lines represent cells incubated with D39 while thick lines represent cells treated with indicated inhibitors followed by incubation with D39. Data from one of two independent experiments are shown. Bars in Panel C and D represent fold increase in Relative Mean Fluorescence Intensity (MFI; mean ± SD) for various groups. ns represents non-significant differences between compared groups.

We next investigated whether the effects observed with PspA would be replicated during infection with *S*. *pneumoniae*. To that end we incubated DCs with wild type D39, strain R6 (derived from D39 that lacks capsule but has PspA) or JY2008 (lacks capsule and surface PspA). As shown in Fig 6A, similar to stimulation with PspA, incubation of DCs with D39 significantly increased PD-L1 surface expression. Interestingly, no significant change in the expression levels of PD-L1 was observed on DCs incubated with JY2008, while incubation of DCs with R6 strain increased the PD-L1 expression to levels comparable with that obtained following stimualtion with D39. These results clearly establish a prominent role for PspA in regulating PD-L1 expression during *S*. *pneumoniae* infection.

We next explored the role for calcium homeostasis in regulating PD-L1 expression during live infection. To that end, DCs were treated with TMB-8 (to inhibit IP_3_R) and EGTA (to inhibit calcium influx from extracellular medium) followed by incubation with *S*. *pneumoniae* D39 and PD-L1 expression was monitored. Similar to the data obtained with PspA (Fig 4) inhibiting internal calcium release completely inhibited *S*. *pneumoniae* mediated upregulation of PD-L1 expression (Fig 6B, left panel), while inhibiting calcium influx from extracellular environment had no significant change in PD-L1 expression (Fig 6B right panel). These data confirm the role of calcium dynamics in regulating PD-L1 expression during *S*. *pneumoniae* infection. Fig 6C and 6D graphically represent the data in Fig 6A and 6B, respectively.

### PspA induced PD-L1 regulates apoptosis

**Fig 7:**
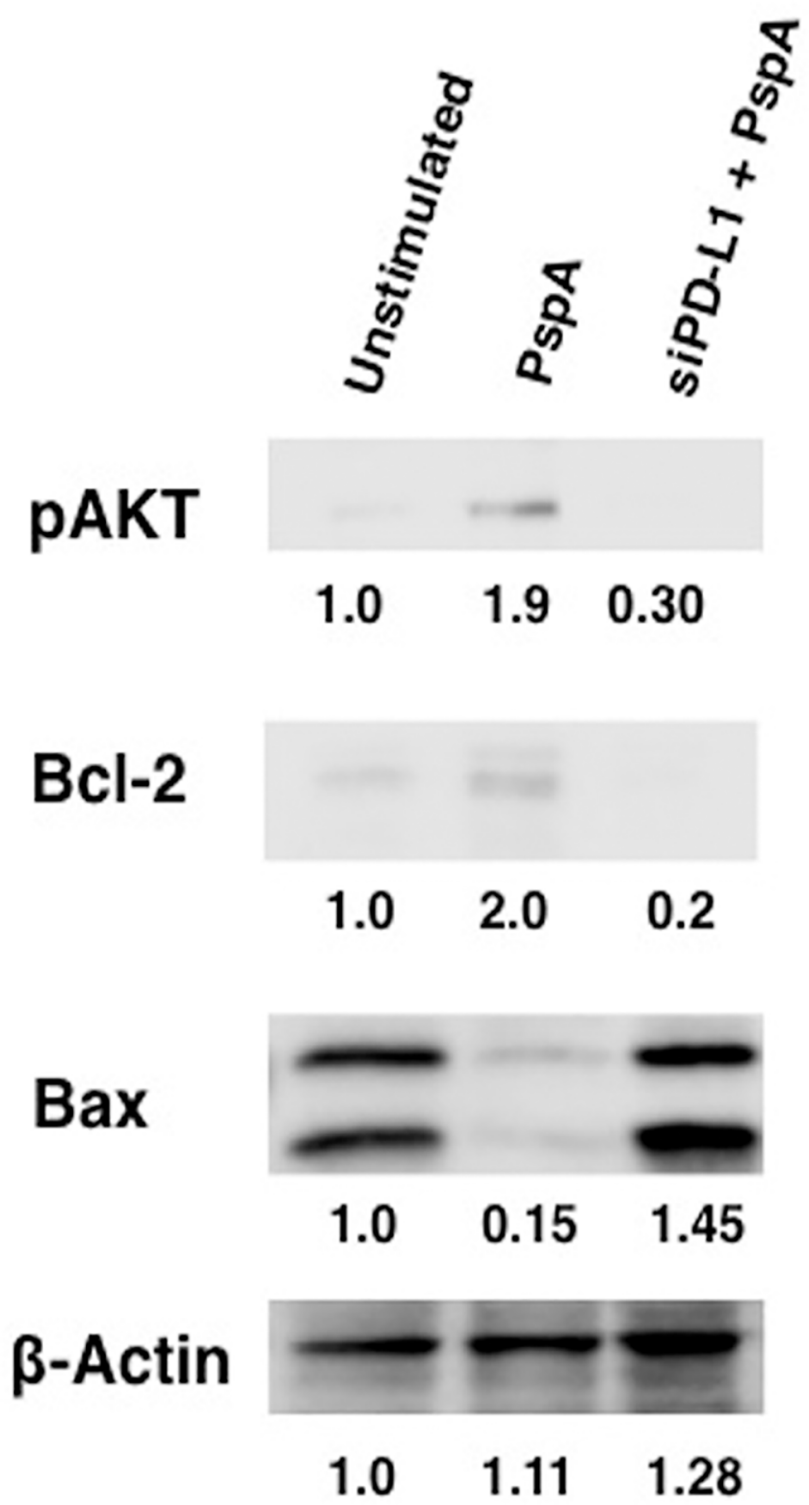
PspA regulates apoptosis of DCs through PD-L1. Mouse bone marrow derived DCs were transfected with siRNAs against PD-L1 for 36h, followed by stimulation with 15 μg/ml PspA for 24h. Cytoplasmic extracts were immunoblotted for indicated molecules. Numbers below the bands represent density of the band relative to unstimulated control. Data from one of two independent experiments are shown.

In the next experiment, we investigated the effects of increased PD-L1 in regulating apoptosis of DCs. DCs were transfected with siRNAs against PD-L1 followed by stimulation with PspA and cytoplasmic levels of pro-apoptotic and anti-apoptotic molecules were determined. As shown in Fig 7, stimulation with PspA increased the levels of anti-apoptotic molecules pAkt and Bcl2, and concomitantly, decreased the levels of pro-apoptotic molecule Bax. However, knockdown of PD-L1 reversed the effects of PspA by significantly reducing the levels of pAkt and Bcl2, and marginally increasing Bax levels relative to β Actin. These results indicate that PspA promoted cell survival of DCs possibly for prolonged survival by increasing the levels of PD-L1. These results point towards a new previously unidentified role for PD-L1 during *S*. *pneumoniae* infection.

## Discussion

With the evolution of more virulent and drug resistant strains of pathogens there is a greater need for deeper investigations into the intricacies of host-pathogen interactions. Successful pathogens like *S*. *pneumoniae* have evolved new strategies to counter existing vaccine and antibiotics [41]. Although multivalent vaccines against *S*. *pneumoniae* infection have been licensed [42], with increased serodiversity of the *S*. *pneumoniae* capsule there is an urgent need to characterize immune evasive strategies and the molecular mechanisms operative therein. One such strategy adopted by many pathogens such as *M*. *tuberculosis*, HIV and other viruses is to inhibit productive immune responses by modulating the expression levels of costimulatory molecules like PD-L1 [43–45].

PD-L1 is a costimulatory molecule that is constitutively expressed on antigen presenting cells like DCs and macrophages. The interaction of PD-1 with PD-L1 induces T cell inhibition and anergy, thereby terminating or preventing a productive T cell response. Detailed investigations into the mechanisms involved in PD-L1 expression during *S*. *pneumoniae* infection or by its antigens have not been carried out. Haas and coworkers have very recently demonstrated the role of PD-1 in *S*. *pneumoniae* survival [46]. PD-1^-/-^ mice or wild type mice pre-treated with blocking antibody to PD-1 displayed increased survival against lethal infection with *S*. *pneumoniae* that involved increased IgG production by pneumococcal capsule-specific B cells [46]. Therefore, in this study we investigated the role of PspA, a key surface molecule of *S*. *pneumoniae* in regulating PD-L1 expression.

Our results indicate that PspA upregulated PD-L1 in a dose and time dependent manner. Characterization of the mechanisms involved in upregulation of PD-L1 expression pointed towards a role for TLR2, wherein, costimulating PspA along with TLR2 synergistically increased PD-L1 expression. TLR2 has generally been shown to induce responses that mediated protection from invading pathogens. For example, TLR2^-/-^ mice are more susceptible to bacterial infections [47]. However, recent reports have pointed towards a dual role for this receptor, that at times induces responses that favour the pathogen. For example, our own recent study indicated a role for TLR2 in positively regulating the expression of voltage gated calcium channels that play an inhibitory role in mediating immune responses to *M*. *tuberculosis* [48]. Further, the 19 kDa lipoprotein of *M*. *tuberculosis* suppresses interferon gamma mediated protection via TLR2 [49]. A study on meningitis model showed increased bacterial level and enhanced disease in TLR2^-/-^ mice [50]. Dissection of the TLR pathway revealed the role for MyD88 dependent pathway in regulating PspA induced PD-L1 expression.

In the next set of experiments we investigated the role of key intermediates of multiple pathways that have been shown to regulate immune responses to infections. Our data pointed towards a crucial role for calcium in PspA mediated PD-L1 expression. Interestingly, not only did calcium regulate PD-L1 expression its routing from different channels induced contrasting effects. While calcium from internal stores positively regulated PD-L1 expression, calcium entry from external medium negatively regulated PD-L1 expression. It is well documented that calcium homeostasis and dynamics play important roles in mediating gene expression [51]. Recently, it has been shown that calcium is required for the activation of human macrophages and IL-1β secretion [52]. Also, increased cytosolic calcium is necessary for the core assembly of HBV virus during infection of HBx [53]. Calcium from different stores could induce differential activation of transcription factors that could in turn induce differential gene expression. Experiments are underway towards this direction. We further characterized the role of calcium by investigating the contribution of molecular sensors of calcium influx in regulating PD-L1 expression. Our data indicate a crucial and positive role for these sensors in PD-L1 expression. These data point towards an unique and unidentified mechanism regulating PD-L1 expression during *S*. *pneumoniae* infection. Further, it has been demonstrated that pneumococcal infection of host epithelial cells induces the release of calcium from intracellular stores in a phospholipase C-dependent manner [54]. Our own work has identified crucial roles for voltage gated calcium channels in negatively regulating protective responses to *M*. *tuberculosis* in DCs and macrophages [55]. Inhibiting voltage gated calcium channels induced protective responses against *M*. *tuberculosis* and mediated clearance of the pathogen from mice. Recently, we have demonstrated the mechanisms that regulated voltage gated calcium channel expression and interestingly, a positive role for TLR2 was observed during voltage gated calcium channel regulation by *M*. *tuberculosis* and its antigen [48]. Inhibiting these channels further enhanced PspA induced PD-L1 expression (data not shown), indicating a broader inhibitory role of these calcium channels in infections.

We had earlier reported a reciprocal regulation between calcium and ROS during *M*. *tuberculosis* infection [48, 55]. Therefore, we next investigated a role for ROS in PD-L1 expression by PspA. Data showed that ROS also positively regulated PD-L1 expression. In addition to regulating pathogenesis of intracellular bacteria and viruses, the generation of ROS has also emerged as a key signaling aspect that regulates immune responses [56]. Therefore, our data are consistent with these reports on the role of ROS during *S*. *pneumoniae* pathogenesis.

We next confirmed whether our results with PspA are in agreement with data obtained with *S*. *pneumoniae* infection. Results not only affirm the same but also indicate a dominant role for PspA in mediating PD-L1 expression as JY2008 strain failed to upregulate PD-L1 on DCs. Also, incubation of DCs with R6 strain increased the PD-L1 expression to those obtained with stimulation with wild type strain, suggesting the prominent role for PspA (Fig 6). Further, the data also confirmed the critical role of calcium dynamics in PD-L1 expression during infection. Finally, in order to investigate whether up-regulation of PD-L1 had any biological consequences, we investigated its role in mediating apoptosis, a key defence process of innate cells to contain infection. Results showed that knocking down PD-L1 promoted apoptosis thus indicating that upregulation of PD-L1 indeed modulated a key function of DCs.

In summary, in this study we have identified a role for *S*. *pneumoniae* and its surface antigen PspA in regulating a key inhibitory costimulatory molecule and also the mechanism involved therein. A novel role for calcium homeostasis has been identified that would improve our understanding of complex interactions that regulate immune responses during *S*. *pneumoniae* infection. Further investigations into the role of SOCE and other molecular sensors in regulating PspA induced immune responses are underway.

## Supporting Information

S1 Fig Commassie blue stained SDS PAGE of recombinant PspA^3–286^ purified by Ni-NTA affinity chromatography. 20 μg of the recombinant protein was loaded. The purity of the recombinant PspA ^3–286^ preparations were found to be 96–98%.
(DOC)

S2 Fig Efficiency of siRNA mediated knockdown of genes. Mouse bone marrow derived DCs were transfected with siRNAs directed against the indicated gene for 36h as described previously [39]. Total RNA was isolated from cells and the relative expression of indicated genes was analyzed by semi-quantitative RT-PCR. Beta actin was used as the reference gene for comparing relative transcript level. Data from one of two independent experiments are shown.
(DOC)

S3 Fig PspA specifically upregulates surface expression of PD-L1. Mouse bone marrow derived DCs were stimulated with 15 μg/ml PspA for 24h and PD-L1 expression was monitored by flow cytometry. Thick line represents PspA stimulated cells stained with isotype matched control monoclonal antibody. Dotted line represents unstimulated cells stained for PD-L1. Thin line represents PspA stimulated cells stained with antibody specific to PD-L1.
(DOC)

S4 Fig TLR2 knockdown restricts the PD-L1 upregulation. Mouse bone marrow derived DCs were transfected with siRNA against TLR2 for 36h, followed by stimulation with PspA for 24h. PD-L1 levels were monitored using flow cytometry. In Panel A, dotted line represents unstimulated cells transfected with control siRNA. Thin lines represent cells transfected with control siRNA followed by stimulation with PspA for 24h. Bold line represents the cells transfected with siRNA specific to TLR2 followed by stimulations with PspA. Data from one of three independent experiments is shown. Panel B, shows the relative expression of PD-L1 represented as relative MFIs. Panel C shows the knockdown efficiency of siRNA against TLR2 in DCs.
(DOC)

S5 Fig PspA increases ROS generation in DCs. Mouse bone marrow derived DCs were stimulated with PspA for 2h. 30 minutes prior to the incubation period cells were loaded with 10 μM DCFH-DA. At the end of incubation period, cells were quickly and thoroughly washed with culture medium and immediately analyzed for ROS level by flow cytometry. Thin line represents unstimulated cells stained for ROS. Bold line represents cells stimulated with PspA (15 μg/ml).
(DOC)
