# Supplementary material for: Pneumococal Surface Protein A (PspA) Regulates Programmed Death Ligand 1 Expression on Dendritic Cells in a Toll-Like Receptor 2 and Calcium Dependent Manner
Source: PLoS One. 2015 Jul 27;10(7):e0133601. doi: 10.1371/journal.pone.0133601 (PMC4516265; doi:10.1371/journal.pone.0133601)
Supplement: S1 Fig — 20 μg of the recombinant protein was loaded. The purity of the recombinant PspA 3–286 preparations were found to be 96–98%. (DOC) [file pone.0133601.s001.doc]

Supporting Information

**
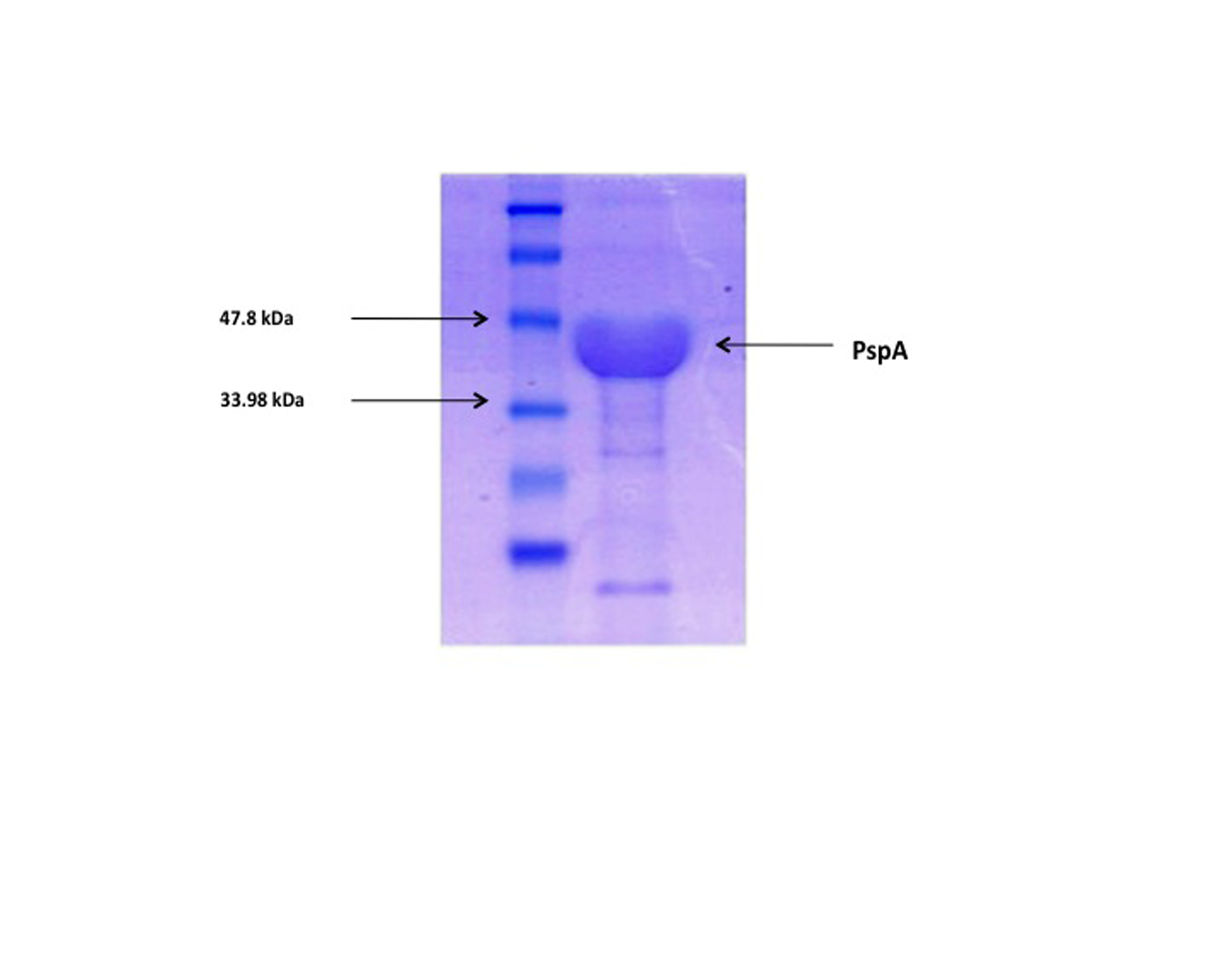
**

**S1 Fig. Coomassie blue stained SDS PAGE (12%) of recombinant PspA^3-286^ purified by Ni-NTA affinity chromatography.** 20 µg of the recombinant protein was loaded. The purity of the recombinant PspA ^3-286^ preparations were found to be 96-98%.
