# Supplementary material for: Pneumococal Surface Protein A (PspA) Regulates Programmed Death Ligand 1 Expression on Dendritic Cells in a Toll-Like Receptor 2 and Calcium Dependent Manner
Source: PLoS One. 2015 Jul 27;10(7):e0133601. doi: 10.1371/journal.pone.0133601 (PMC4516265; doi:10.1371/journal.pone.0133601)
Supplement: S2 Fig — Mouse bone marrow derived DCs were transfected with siRNAs directed against the indicated gene for 36h as described previously [39]. Total RNA was isolated from cells and the relative expression of indicated genes was analyzed by semi-quantitative RT-PCR. Beta actin was used as the reference gene for comparing relative transcript level. Data from one of two independent experiments are shown. (DOC) [file pone.0133601.s002.doc]

**
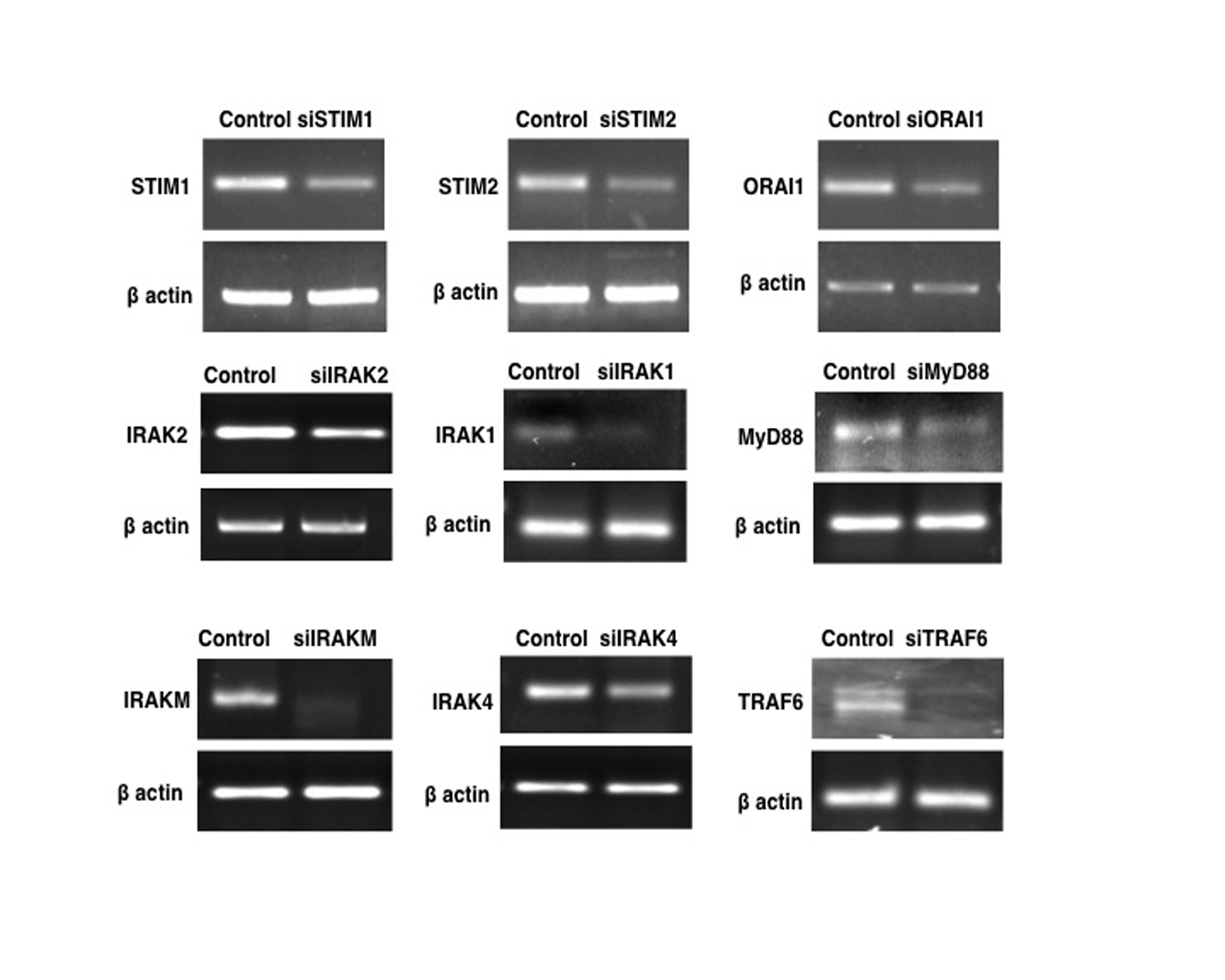
**

**S2 Fig. Efficiency of siRNA mediated knockdown of genes**. Mouse bone marrow derived DCs were transfected with siRNAs directed against the indicated gene for 36h as described previously [39]. Total RNA was isolated from cells and the relative expression of indicated genes was analyzed by semi-quantitative RT-PCR. Beta actin was used as the reference gene for comparing relative transcript level. Data from one of two independent experiments are shown.
