# Supplementary material for: Pneumococal Surface Protein A (PspA) Regulates Programmed Death Ligand 1 Expression on Dendritic Cells in a Toll-Like Receptor 2 and Calcium Dependent Manner
Source: PLoS One. 2015 Jul 27;10(7):e0133601. doi: 10.1371/journal.pone.0133601 (PMC4516265; doi:10.1371/journal.pone.0133601)
Supplement: S3 Fig — Mouse bone marrow derived DCs were stimulated with 15 μg/ml PspA for 24h and PD-L1 expression was monitored by flow cytometry. Thick line represents PspA stimulated cells stained with isotype matched control monoclonal antibody. Dotted line represents unstimulated cells stained for PD-L1. Thin line represents PspA stimulated cells stained with antibody specific to PD-L1. (DOC) [file pone.0133601.s003.doc]

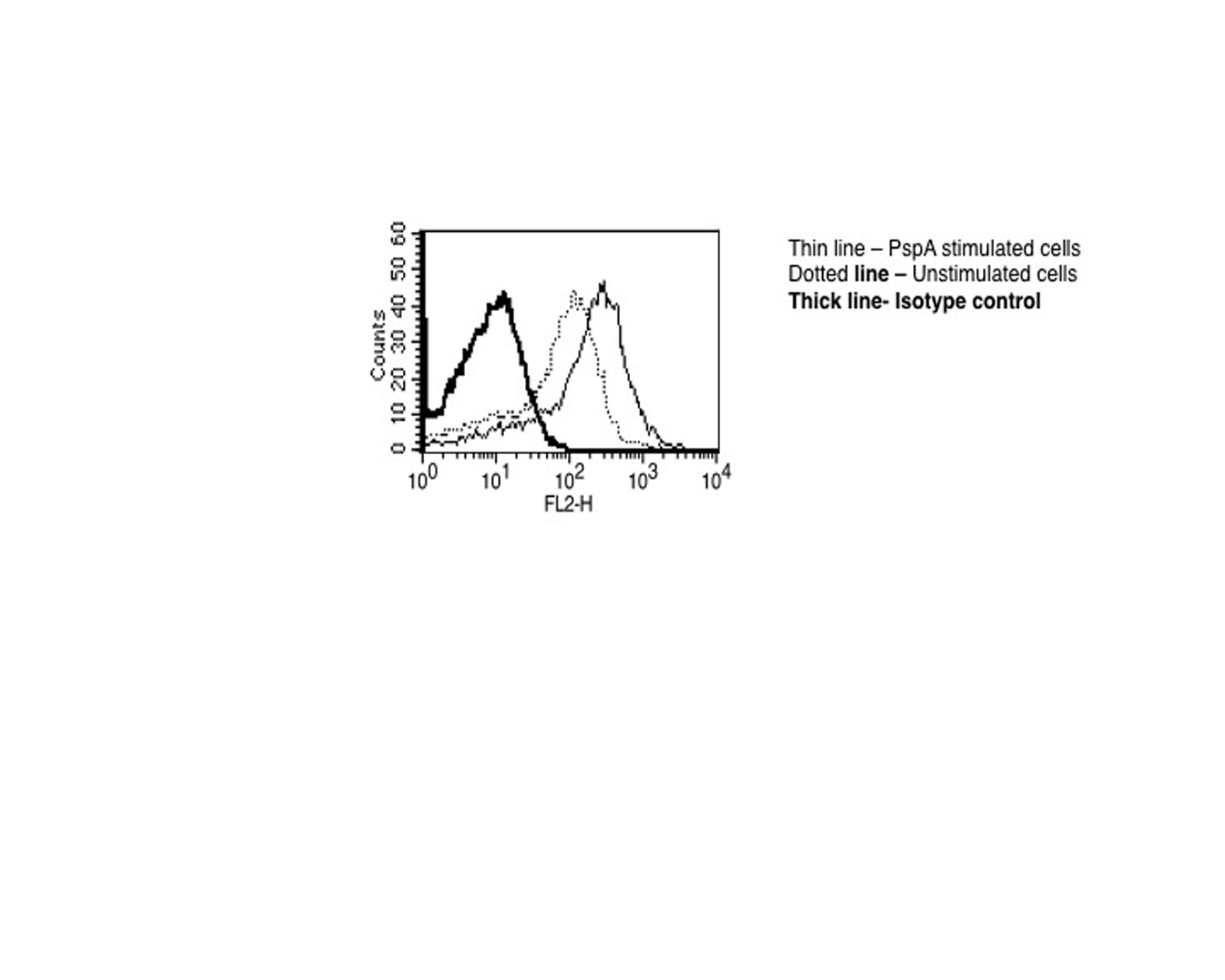


**S3 Fig. PspA specifically upregulates surface expression of PD-L1.** Mouse bone marrow derived DCs were stimulated with 15 μg/ml PspA for 24h and PD-L1 expression was monitored by flow cytometry. Thick line represents PspA stimulated cells stained with isotype matched control monoclonal antibody. Dotted line represents unstimulated cells stained for PD-L1. Thin line represents PspA stimulated cells stained with antibody specific to PD-L1.
