# Supplementary material for: Pneumococal Surface Protein A (PspA) Regulates Programmed Death Ligand 1 Expression on Dendritic Cells in a Toll-Like Receptor 2 and Calcium Dependent Manner
Source: PLoS One. 2015 Jul 27;10(7):e0133601. doi: 10.1371/journal.pone.0133601 (PMC4516265; doi:10.1371/journal.pone.0133601)
Supplement: S4 Fig — Mouse bone marrow derived DCs were transfected with siRNA against TLR2 for 36h, followed by stimulation with PspA for 24h. PD-L1 levels were monitored using flow cytometry. In Panel A, dotted line represents unstimulated cells transfected with control siRNA. Thin lines represent cells transfected with control siRNA followed by stimulation with PspA for 24h. Bold line represents the cells transfected with siRNA specific to TLR2 followed by stimulations with PspA. Data from one of three independent experiments is shown. Panel B, shows the relative expression of PD-L1 represented as relative MFIs. Panel C shows the knockdown efficiency of siRNA against TLR2 in DCs. (DOC) [file pone.0133601.s004.doc]

**
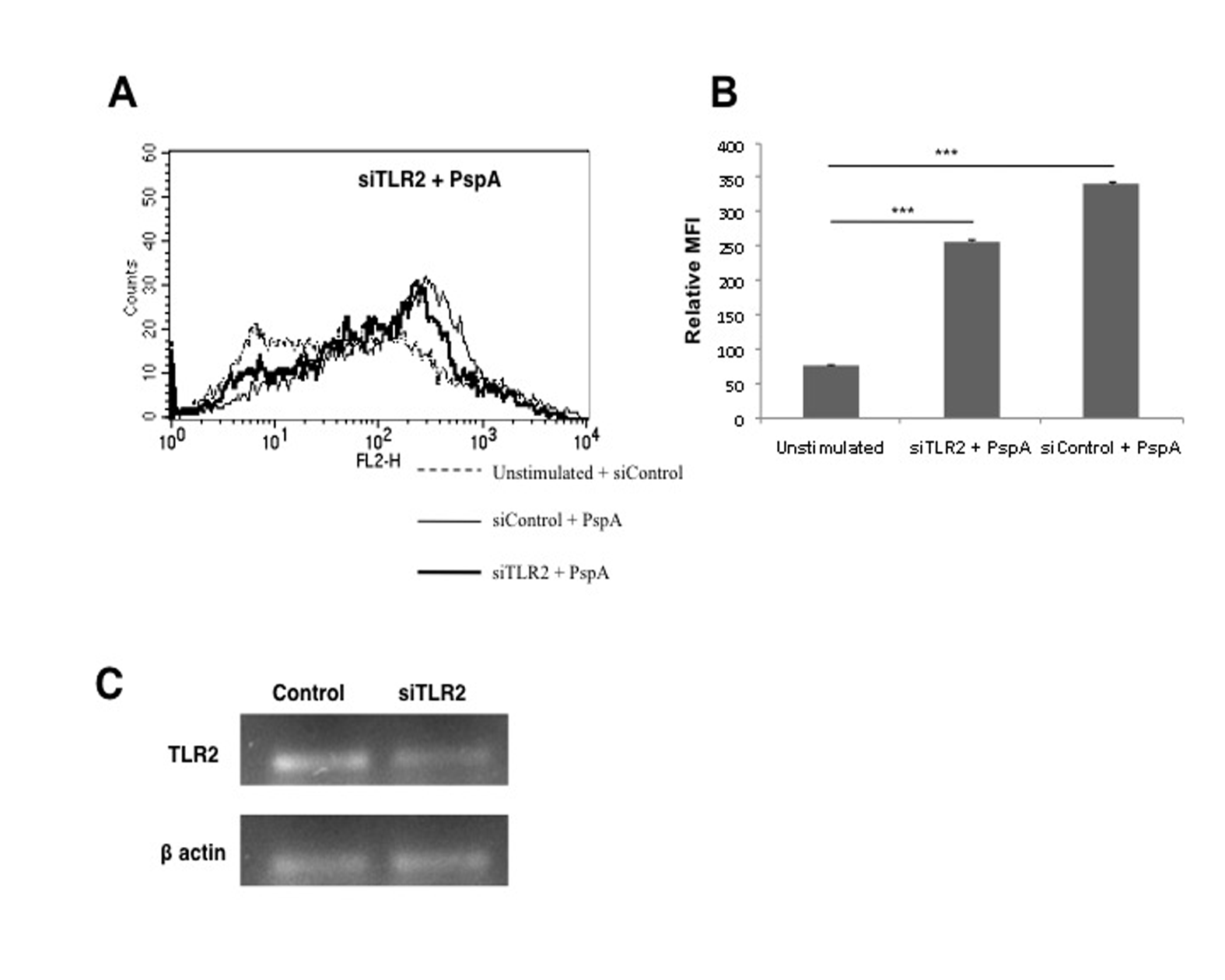
**

**S4 Fig. TLR2 knockdown restricts the PD-L1 upregulation.** Mouse bone marrow derived DCs were transfected with siRNA against TLR2 for 36h, followed by stimulation with PspA for 24h. PD-L1 levels were monitored using flow cytometry. In Panel A, dotted line represents unstimulated cells transfected with control siRNA. Thin lines represent cells transfected with control siRNA followed by stimulation with PspA for 24h. Bold line represents the cells transfected with siRNA specific to TLR2 followed by stimulations with PspA. Data from one of three independent experiments is shown. Panel B, shows the relative expression of PD-L1 as relative MFIs. Panel C shows the knockdown efficiency of siRNA against TLR2 in DCs.
