# Supplementary material for: Pneumococal Surface Protein A (PspA) Regulates Programmed Death Ligand 1 Expression on Dendritic Cells in a Toll-Like Receptor 2 and Calcium Dependent Manner
Source: PLoS One. 2015 Jul 27;10(7):e0133601. doi: 10.1371/journal.pone.0133601 (PMC4516265; doi:10.1371/journal.pone.0133601)
Supplement: S5 Fig — Mouse bone marrow derived DCs were stimulated with PspA for 2h. 30 minutes prior to the incubation period cells were loaded with 10 μM DCFH-DA. At the end of incubation period, cells were quickly and thoroughly washed with culture medium and immediately analyzed for ROS level by flow cytometry. Thin line represents unstimulated cells stained for ROS. Bold line represents cells stimulated with PspA (15 μg/ml). (DOC) [file pone.0133601.s005.doc]

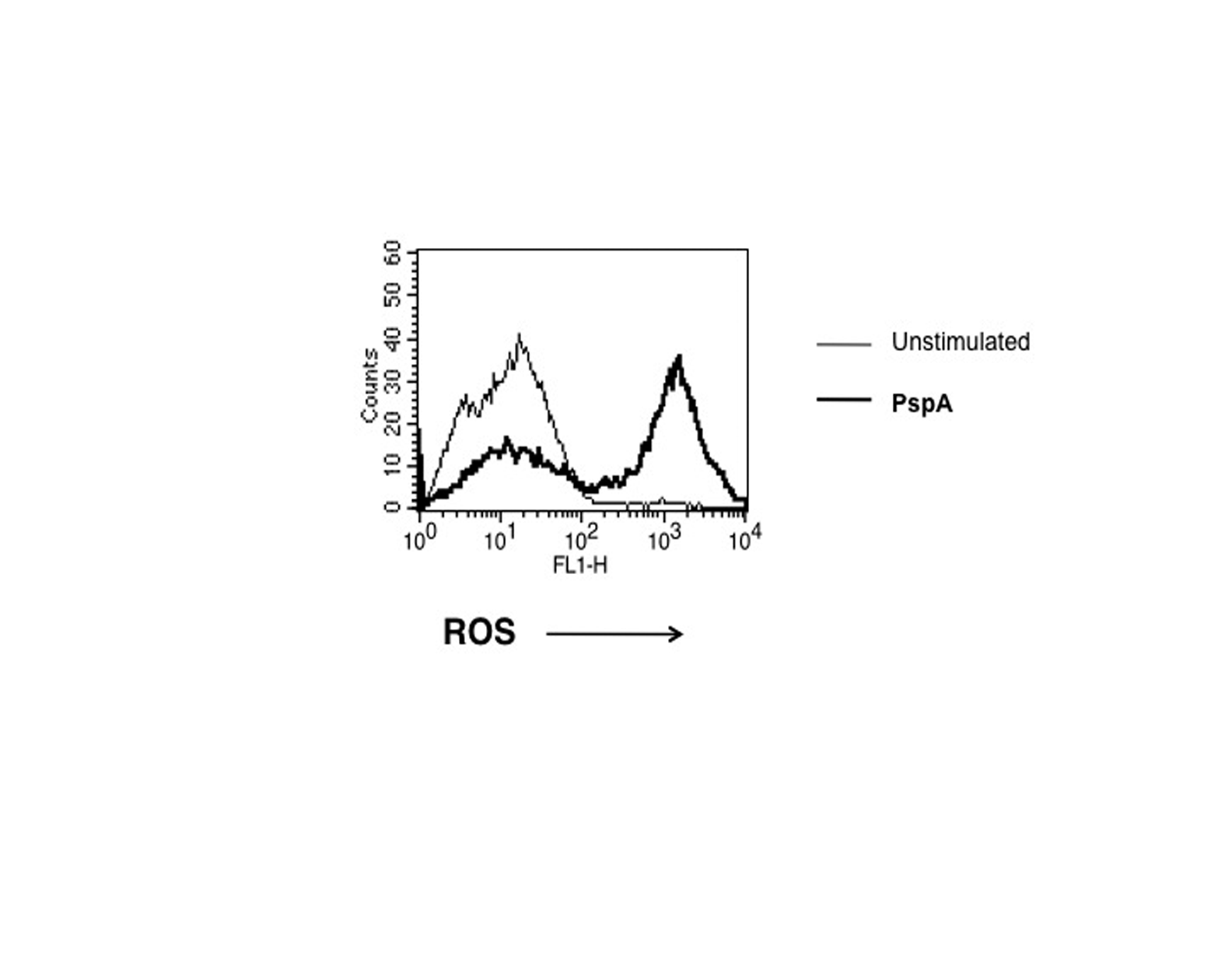


**S5 Fig. PspA increases ROS generation in DCs**. Mouse bone marrow derived DCs were stimulated with PspA for 2h. 30 minutes prior to the incubation period cells were loaded with 10 μM DCFH-DA. At the end of incubation period, cells were quickly and thoroughly washed with culture medium and immediately analyzed for ROS level by flow cytometry. Thin line represents unstimulated cells stained for ROS. Bold line represents cells stimulated with PspA (15 μg/ml).
